# Machine Learning-Based Stator Current Data-Driven PMSM Stator Winding Fault Diagnosis

**DOI:** 10.3390/s22249668

**Published:** 2022-12-10

**Authors:** Przemyslaw Pietrzak, Marcin Wolkiewicz

**Affiliations:** Department of Electrical Machines, Drives and Measurements, Wroclaw University of Science and Technology, Wybrzeze Wyspianskiego 27, 50-370 Wroclaw, Poland

**Keywords:** permanent magnet synchronous motor, fault diagnosis, condition monitoring, interturn short circuits, artificial intelligence, machine learning, short-time Fourier transform

## Abstract

Permanent magnet synchronous motors (PMSMs) have become one of the most important components of modern drive systems. Therefore, fault diagnosis and condition monitoring of these machines have been the subject of many studies in recent years. This article presents an intelligent stator current-data driven PMSM stator winding fault detection and classification method. Short-time Fourier transform is applied in the process of fault feature extraction from the stator phase current symmetrical components signal. Automation of the fault detection and classification process is carried out with the use of three selected machine learning algorithms: support vector machine, naïve Bayes classifier and multilayer perceptron. The concept and online verification of the original intelligent fault diagnosis system with the potential of a real industrial deployment are demonstrated. Experimental results are presented to evaluate the effectiveness of the proposed methodology.

## 1. Introduction

In recent years, permanent magnet synchronous motors (PMSMs) have become increasingly popular in industrial applications [1]. This fact is associated with their high efficiency, high power density and excellent dynamic performance. These properties make the use of PMSMs in drive systems perfectly in line with the current trend of sustainable development in the industry, as it allows to meet the requirements of using highly efficient, energy-saving and environmentally friendly solutions. Moreover, significant progress in the field of microprocessor technology, power electronics and materials engineering for permanent magnets contributed to the notable popularization of PMSM drives in the robotics, automotive, transport, home appliances and aviation industries [2]. Nonetheless, even when operated in a normal environment and under rated conditions, PMSMs are exposed to various types of damage.

Taking into account the growing popularity of PMSMs, fault diagnosis and monitoring of the condition of these machines have also become an important issue. The complex and demanding operating environment, such as high temperature, vibration and humidity, makes the PMSMs even more vulnerable to failures [3]. All of the faults can lead to the interruption of the PMSM drive system operation and unexpected maintenance breaks in processes if not detected in time. Therefore, in recent years, PMSMs fault diagnosis and condition monitoring have attracted many studies [4,5].

PMSMs failures can be divided according to their type into mechanical, magnetic and electrical damages [6]. Electrical damages are mainly stator winding faults. According to the IEEE and EPRI statistics [7,8], stator winding faults represent between 36% and 66% of all electric motor failures, depending on the type and size of the machine. It makes them one of the most common faults of AC motors [9].

Stator winding faults have a very destructive character. They begin mainly as an imperceptible short circuit of single turns—interturn short circuit (ITSC) and then spread very quickly to the entire winding, leading to the phase-to-phase or phase-to-ground short circuit. ITSCs are often caused by damage of the stator winding insulation. Insulation damage results from abrasion caused by mechanical stress or overheating of the winding as a result of too high loads on the motor [10]. If these faults are not detected and diagnosed in time, they can cause emergency stops of the technological process, safety accidents and significant financial losses [11]. Moreover, ITSCs may cause irreversible demagnetization of permanent rotor magnets [12].

Due to their destructive nature, ITSCs are considered one of the most difficult to detect failures in AC motors. Standard safety circuits used nowadays in industrial drive systems do not react to the short circuiting of several turns in a phase, because it causes too many small changes in the amplitudes of the phase currents. Therefore, new methods are still being sought, based on the measurement and processing of diagnostic signals, allowing for real-time monitoring of motors condition and alerting the user in the initial stage of failure [5].

The requirements resulting from the idea of Industry 4.0, and also the growing number of drive systems in which more and more operating parameters are registered, lead to an increasing emphasis on the diagnosis applications for electric drive systems, especially drives that utilize high-efficient PMSMs [13]. Modern fault diagnosis systems should provide automatic inference about the condition of the motor. To meet this requirement, these systems should consist of at least two main modules: the first one, responsible for the acquisition and processing of the diagnostic signals in order to extract the fault symptoms, and the second one, which, on the basis of raw diagnostic signals or the extracted symptoms, provides automatic inference about the state of the motor.

Over the years, several methods have been developed for the extraction of PMSM stator winding fault symptoms. These methods are based on signal analysis using various signal processing approaches. They are currently of fundamental importance in the diagnosis of electric motors [14,15,16]. The most frequently used signal in the diagnosis of stator winding faults is the stator phase current, its measurement of which is noninvasive and easy to implement in any drive system [17,18]. Moreover, stator winding faults are also well reflected in voltage [19] and axial flux [20] signals as well as the internal signals of the control structure of the PMSM drive system [21].

The characteristic symptoms (features) extraction methods can be classified into three main groups: time domain methods, frequency domain methods and time-frequency methods. Time domain methods are mainly based on statistical analysis of the signal. They use signal parameters, such as mean, peak, kurtosis and mean square for the condition monitoring of the motor [22]. However, due to the limitations of the methods belonging to this group, they are not very common in PMSM fault diagnosis.

The most popular ITSC fault diagnosis methods are methods that perform frequency domain analyses, especially spectral analysis of the diagnostic signal using a Fast Fourier Transform (FFT) [23]. The analysis of the amplitudes of components in the FFT current spectrum is well known in the literature as Motor Current Signature Analysis (MCSA). It is based on the monitoring of changes in the amplitude values of individual frequency components in the FFT spectrum as a result of a fault [23]. The effectiveness of this method for the detection of PMSM stator winding faults has been confirmed, among others, in [24]. The improvement of MCSA with the stator phase current Extended Park’s Vector Analysis (EPVA) is presented in [25]. Nevertheless, in recent years, due to the increasing computing power of the microcontroller-based embedded systems, the use of more advanced frequency domain signal processing methods that are based on high order statistics has gained popularity. These methods are called High Order Transforms (HOTs). HOTs, such as MUltiple SIgnal Classification (MUSIC) and bispectrum, have also been applied to the PMSM stator winding fault diagnosis in [26] and [27], respectively. However, these methods also have some limitations. The main limitation is the lack of information about the time of occurrence of a given frequency component and, in most cases, the need for a long measurement time to achieve high symptoms extraction effectiveness. Methods that perform a time–frequency analysis do not have such limitations. 

The time-frequency domain methods provide the location in time while simultaneously capturing the frequency information. This group of methods includes, among others, the Continuous Wavelet Transform (CWT) [28], the Hilbert–Huang transform [29] and Short-Time Fourier Transforms (STFT). The STFT is one of the most popular time-frequency analyses that have been used in the diagnostics of electric motor failures. However, its application in the past has been mainly studied for induction motors [30,31].

All of the above-mentioned methods can be used in the module of the fault diagnosis system that is responsible for the symptoms extraction. However, this is not enough for modern condition monitoring and fault diagnosis systems. There is a significant need to automate the process of inferring the state of the motor. To meet these requirements, Machine Learning (ML) algorithms are increasingly used. The main task of ML algorithms in the fault diagnosis domain is to fully automate the fault detection and classification process based on the input data obtained from the diagnostic signal analysis. 

Over the years, much research has been devoted to fault detectors and classifiers that are based on ML algorithms. They can be divided into classical ML algorithms and those inspired by the human brain operation principle—artificial neural networks (ANNs). Classical ML algorithms include K-Nearest Neighbors (KNN) [32], Support Vector Machine (SVM) [33], Naïve Bayes (NB) and Decision Tree (DT). Among ANNs, the neural networks with a classical (shallow) structure and those based on deep learning (DL) can be distinguished. The MultiLayer Perceptron (MLP) is one of the most commonly used ANN types in an electric motor fault diagnosis [34,35,36]. The Radial Basis Function (RBF) ANN and Self Organizing Maps (SOMs) are also verified in this field of research [37,38,39]. In recent years, usage of DL-based neural networks—Deep Neural Networks (DNNs)—is very popular. Among the different types of DNNs, Convolutional Neural Networks (CNNs) are especially effective. Nevertheless, DNN structures require higher computing power than classical ML algorithms. On the other hand, CNNs allow for very effective fault detection based on a raw diagnostic signal without the signal processing stage, which significantly shortens the detection time, but requires a longer training time of the CNN model and a complex network structure [40]. 

Nowadays, it is also popular to combine advanced signal processing algorithms, the result of which is presented as an image, with CNN. This allows for the reduction of training time and the use of a simpler network structure while achieving high efficiency [41,42,43]. Nonetheless, as opposed to detecting faults of induction motors and mechanical damages of PMSMs, there are still few scientific papers in which the usage of simple ML algorithms, such as SVM and NB, to detect PMSM stator winding faults is verified, especially taking into account the analysis of the impact of the key parameters of fault classifiers on their effectiveness.

The main goal of this article is to verify the possibility of using the STFT analysis of stator phase current symmetrical components to extract the symptoms of ITSCs in the PMSM stator winding and selected ML algorithms (SVM, NB and MLP) for the automatic detection and classification of this type of failure. The contributions and original elements of this paper can be summarized as follows:

(1) Application of the STFT analysis of the positive and negative stator phase currents symmetrical component to extract symptoms of the ITSC in the PMSM stator winding, verified in a wide range of load torques and power supply frequencies (rotation speed).
(2) Development and verification of the effectiveness of the hybrid diagnostic methods that combines the STFT analysis of the stator phase currents symmetrical components and selected ML algorithms (SVM, NB and MLP) for ITSC detection at the early stage of the fault.
(3) Detailed analysis of the influence of key parameters (hyperparameters) of selected ML algorithms on the accuracy of fault classification. The improvement of the classifiers effectiveness by properly tuning the model and training parameters is shown.
(4) The proposal and online verification of the intelligent PMSM stator winding fault diagnosis system developed in the LabVIEW and MATLAB programming environment. The developed data-driven intelligent system has significant potential for real deployment in the industry.

The rest of the paper is organized as follows. After the introduction, Section 2 presents the impact of the stator winding fault on the waveforms of stator phase currents symmetrical components. Section 3 gives the theoretical basis of the STFT. The experimental setup is presented in Section 4. In Section 5, the stator winding fault symptoms extraction part with the use of STFT analysis is presented. The theoretical basis and training process of the selected ML based stator winding fault classifier models is presented in Section 6. In Section 7, the concept and online verification of the intelligent diagnosis system of the PMSM stator winding faults are shown. Finally, Section 8 contains conclusions resulting from the results obtained. 

## 2. Impact of the PMSM Stator Winding Fault on the Waveforms of Symmetrical Components of the Stator Phase Currents

The asymmetry of the stator phase currents caused by the ITSCs in the PMSM stator winding has an impact on the values of the stator currents symmetrical components. Since there is no zero sequence component in three-phase PMSMs, only positive and negative sequence components are calculated using the following equation [44]:

(1) $$\left[\begin{array}{c} I_{1}\\ I_{2} \end{array}\right]=\frac{1}{3}\left[\begin{array}{l} \begin{array}{ccc} 1 & a & a^{2} \end{array}\\ \begin{array}{ccc} 1 & a^{2} & a \end{array} \end{array}\right]\cdot \left[\begin{array}{l} I_{sA}\\ I_{sB}\\ I_{sC} \end{array}\right]$$

where:

*I*_1_, *I*_2_—positive and negative stator phase current component in steady state,

*I_sA_*, *I_sB_*, *I_sC_*—stator phase currents in steady state,

and:

(2) $$a=e^{j\frac{2\pi }{3}}$$

Equation (1) applies to the sinusoidal signals of the stator phase currents in a steady state. Nonetheless, PMSMs are supplied by Voltage Source Inverters (VSIs), which introducs a number of additional harmonics, causing the distortion of voltages and currents. In such cases, in order to use the classical method of symmetrical components calculation, it is necessary to filter out the disturbing harmonics or extract only the fundamental component of the supply voltage (*f_s_*). In this paper, the second approach is used. It is based on the calculation of instantaneous values of the stator current symmetrical components using the 90° shift operator in the time domain, according to [44]:

(3) $$\left[\begin{array}{l} i_{1}\\ i_{2} \end{array}\right]=\frac{1}{3}\left[\begin{array}{c} i_{sA}-\frac{1}{2}(i_{sB}+i_{sC})+\frac{\sqrt{3}}{2}S_{90}(i_{sB}-i_{sC})\\ i_{sA}-\frac{1}{2}(i_{sB}+i_{sC})-\frac{\sqrt{3}}{2}S_{90}(i_{sB}-i_{sC}) \end{array}\right]$$

where:

*i*_1_, *i*_2_—instantaneous value of the positive and negative sequence stator phase current component,

*i_sA_*, *i_sB_*, *i_sC_*—instantaneous value of the stator current in phase A, B and C,

*S*_90_—operator of a phase shift by an angle of 90° in the time domain.

**Figure 1:**
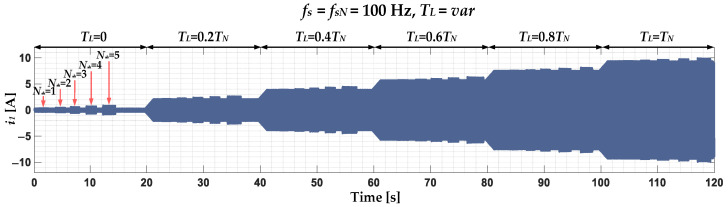
Impact of ITSC in the PMSM stator winding on the waveform of the positive sequence component of the stator phase currents, experimental study (*T_L_* = var, *f_s_* = *f_sN_* = 100 Hz).

The influence of the ITSCs on the PMSM stator winding on the positive stator phase current sequence component waveform for the nominal power supply frequency *f_s_* (rotation speed), different load torque *T_L_* levels and number of shorted turns *N_sh_* is presented in Figure 1. As shown, for each of the *T_L_* set—in the range (0–1)*T_N_* with 0.2 *T_N_* step—momentary short circuits of 1 to 5 successive turns in phase A of the PMSM stator winding are performed. Analysis of this waveform shows that the amplitude changes of the stator current positive sequence component as a result of ITSC is noticeable, but the influence of the *T_L_* level is much more significant.

**Figure 2:**
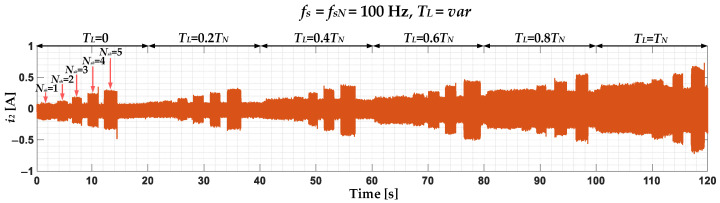
Impact of ITSC in the PMSM stator winding on the waveform of the negative sequence component of the stator phase currents, experimental study (*T_L_* = var, *f_s_* = *f_sN_* = 100 Hz).

The unbalance of the PMSM stator phase currents caused by the ITSC are also visible in the negative sequence component [32]. The waveform of this component for the nominal *f_s_*, different *T_L_* and *N_sh_* is presented in Figure 2. The level of *T_L_* has a much smaller impact on the value of the negative sequence amplitude compared to the positive sequence component, and more importantly, a significant increase resulting from the ITSCs is visible. It can also be observed that the higher the *N_sh_*, the greater the increase in amplitude. 

However, based only on the raw waveforms of the stator phase current positive and negative sequence components, an effective diagnosis and classification of the stator winding fault would be difficult, because for the higher *T_L_* levels (*T_L_* = 0.8*T_N_*, *T_L_* = *T_N_*), the increase caused by the ITSC of a lower number of turns (*N_sh_* = 1, *N_sh_* = 2) is insufficient. Due to the destructive nature and high dynamics of the PMSM stator winding fault, it is necessary to detect this type of damage at the earliest possible stage. Therefore, in this study, the signal processing method (STFT) is used to extract the more sensitive symptoms (features) of the ITSC fault, also in the initial stage of the damage.

## 3. Short-Time Fourier Transform

The frequency domain representation of the signal provided by the classical FFT-based spectral analysis does not contain information about the occurrence of a particular frequency over time. In the field of motor fault diagnosis, information about the fault time can be very useful. Based on this information, the source of the failure can be found. 

The STFT overcomes the limitations of the FFT analysis. It is an extension of FFT for time-frequency domain analysis. To achieve this, the analyzed signal is divided in the time domain through temporary windows of the same width, and subsequently frequency content of each of these windows is obtained using the FFT. The size of the time window defines the resolution of time and frequency [30]. An additional advantage of STFT is its suitability for the analysis of nonstationary signals [45].

In the implementation of the STFT, a design trade-off must be made between the time and frequency resolution. A short window provides good time resolution at the expense of poor frequency resolution and vice versa. The STFT calculates the Fourier Transform (FT) of a function *f*(*t*) over a symmetrical and real window function *w*(*t*), which is translated by time *t* and modulated at frequency *ω*. The continuous domain expression of the STFT is illustrated by [46]:

(4) $$S(t,\omega )=\int_{-\infty }^{\infty }f(t)w(\tau -t)e^{-j\omega \tau }d\tau$$

The magnitude of the STFT yields the spectrogram. In this investigation, the amplitudes of the spectrogram are analyzed. The spectrogram is the result of calculating the frequency spectrum of windowed signal frames. It is a three-dimensional plot of the energy of the signal frequency content as it changes over time and is expressed as follows:

(5) $$spectrogram(t,\omega )=|S(t,\omega ){|}^{2}$$

In the real world, signals are sampled with a fixed sampling frequency (*f_p_*), and the FFT is computed to analyze the frequency spectrum of the signal. Therefore, Equation (4) in the discrete domain is expressed by the following equation [46]:

(6) $$S_{D}[m,k]=\sum_{n=0}^{n=N-1}x[n]w[n-mH]e^{-j\frac{2\pi nk}{N}}$$

where:

*N*—number of FFT points,

*n*—time domain input sample index,

*x*[*n*]—input sample,

*w*[*n*]—window function,

*H*—window size (width),

*k*—frequency index.

The key parameters of the STFT analysis that influence its result are as follows [46]:

- **Sampling frequency (*f_p_*):** It affects the time and frequency resolution of the STFT output. Higher *f_p_* results in better time and frequency resolution and vice versa. In this research, *f_p_* of the STFT-based ITSC symptoms extraction algorithm is set to 8192 Hz, which is typically used in modern drive systems for current measurements.
- **Number of input samples (*N_t_*):** It is the total number of samples of the input signal on which the windowing function is applied. For the 10 s measurement time and *f_p_* = 8192 Hz, the number of input samples is equal to 81920.
- **Window size (*H*):** The window size is responsible for the STFT output resolution in the time domain. The lower the size of the window, the better the resolution in the time domain. In this article, *H* is chosen to be 2048 samples, which is equivalent to the time resolution of 0.25 s. For some applications, the windows are also often overlapped.
- **Type of window function (*w*[*n*])** Rectangular, Triangular, Hanning, Hamming and Barlett are the most popular window functions available to perform STFT. In this research, the Hamming window function is used.

**Figure 3:**
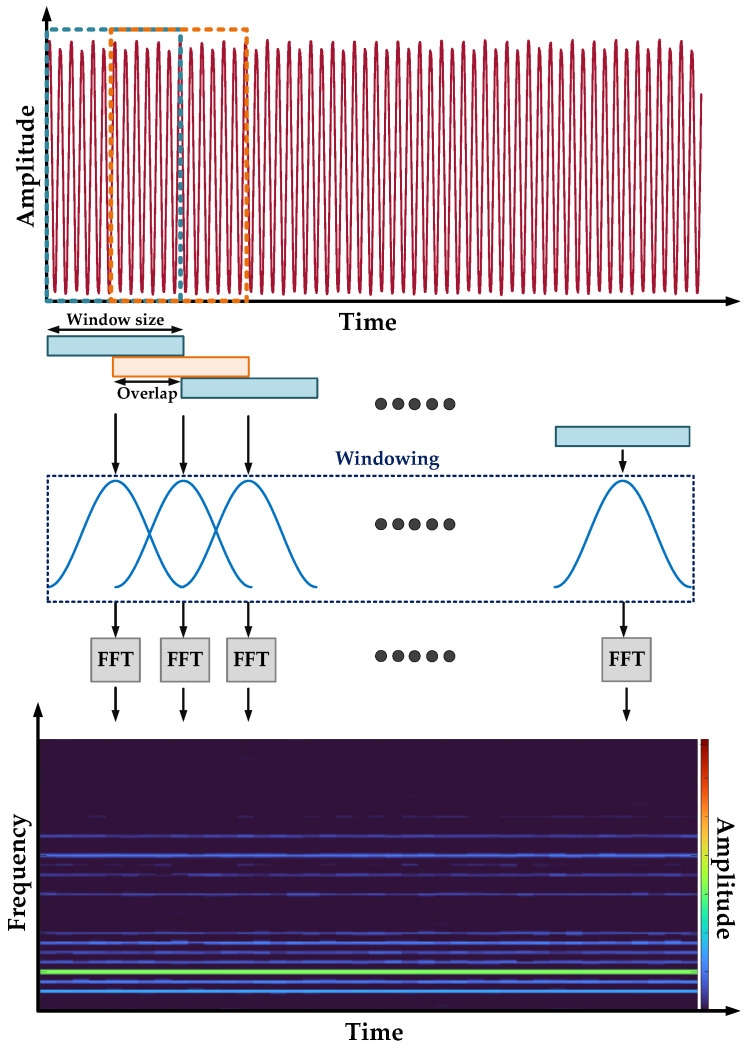
Illustrative presentation of Short-time Fourier transform of a time domain signal.

Figure 3 shows in an illustrative way how the spectrogram of the time domain signal is obtained with the use of the STFT analysis.

## 4. Experimental Setup

**Figure 4:**
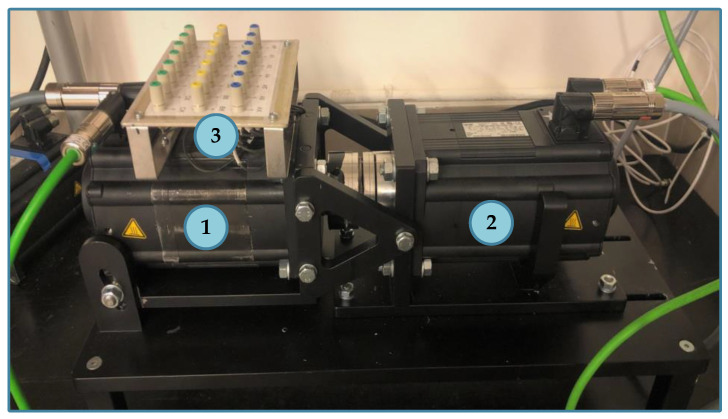
Real view of the experimental stand with (1) tested PMSM, (2) load PMSM and (3) board with the derived phase terminals of the stator winding.

**Figure 5:**
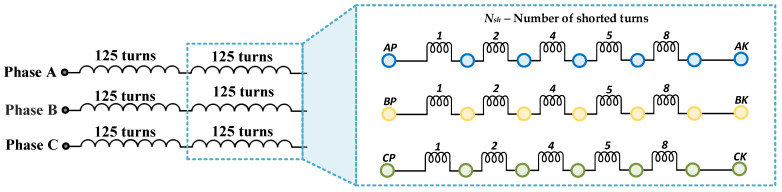
Schematic diagram of the PMSM stator winding phase terminals.

The main part of the experimental setup is a 2.5 kW PMSM supplied from a VSI and operating in the Field-Oriented Control (FOC). The loading machine is a second PMSM with nominal power equal to 4.7 kW. The real view of the laboratory stand is shown in Figure 4. The stator winding construction of the tested PMSM is specially prepared to allow for the physical modeling of the ITSCs of a selected number of turns in a phase. Each of the three phases of the stator winding consists of two coils, at 125 turns each. An illustrative diagram of the derived phase terminals of the tested PMSM stator winding is presented in Figure 5. During the experimental verification, a maximum of five turns in Phase A were short circuited, which accounted for 2% of all turns in one phase. The direct short circuits are performed by connecting the taps corresponding to the given number of turns led out on the terminal board with a wire without limiting the current in the ITSC loop with an additional resistance. The rated parameters of the tested motor are grouped in Table A1 in Appendix A.

**Figure 6:**
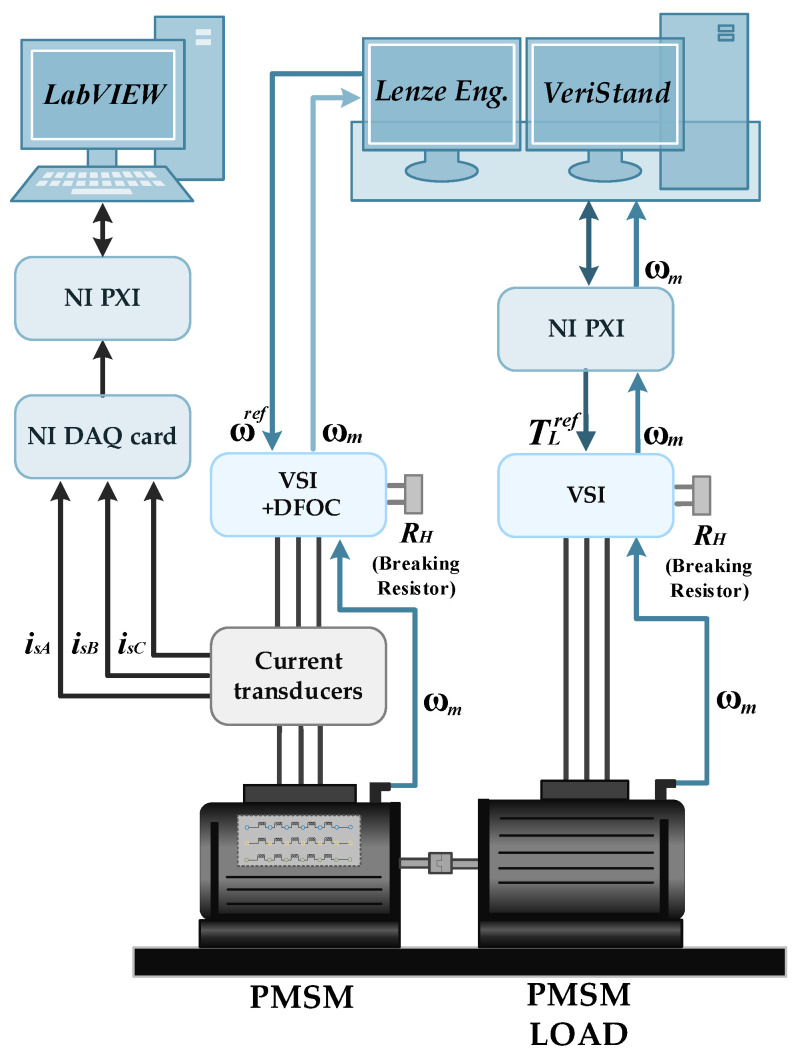
Block diagram of the experimental setup.

The stator phase currents are measured with LEM LA 25-NP multirange current transducers and are transferred to the data acquisition system, which is the National Instruments (NI) DAQ NI PXI-4492 measurement card. This measurement card is placed inside the industrial PC—NI PXI 1082. The diagnostic application is developed in a LabVIEW and MATLAB programming environment. Lenze Engineer software is used to control the tested PMSM, whereas VeriStand is used to set the load torque. The general block diagram of the experimental stand is shown in Figure 6.

## 5. Stator Winding Fault Features Extraction

In this research, the ITSCs symptom extraction process is realized using STFT analysis. The analyzed signals are positive and negative sequence components of the stator phase currents. In the diagnosis of electric motor faults, the application of the STFT is associated with the search for the frequency components that are sensitive to the specific fault. 

As previously mentioned, proper selection of the STFT window width *H* is essential to provide efficient fault symptoms extraction. Nevertheless, there is no single rule for selecting this value. The appropriate *H* value depends on the nature of the analyzed signal, measurement parameters and the specific application. In this research, the window width is set to 2048. The sampling frequency of the signal is 8196 Hz. The selection of *H* = 2048 allows for obtaining a sufficient resolution (0.25 s) in the time domain. The appropriate time domain resolution is extremely important in the diagnosis of the stator winding faults, because they have to be detected as fast as possible. Due to this, too wide a window (the number of samples to be collected for one cycle of STFT analysis) would delay the detection of the fault.

**Figure 7:**
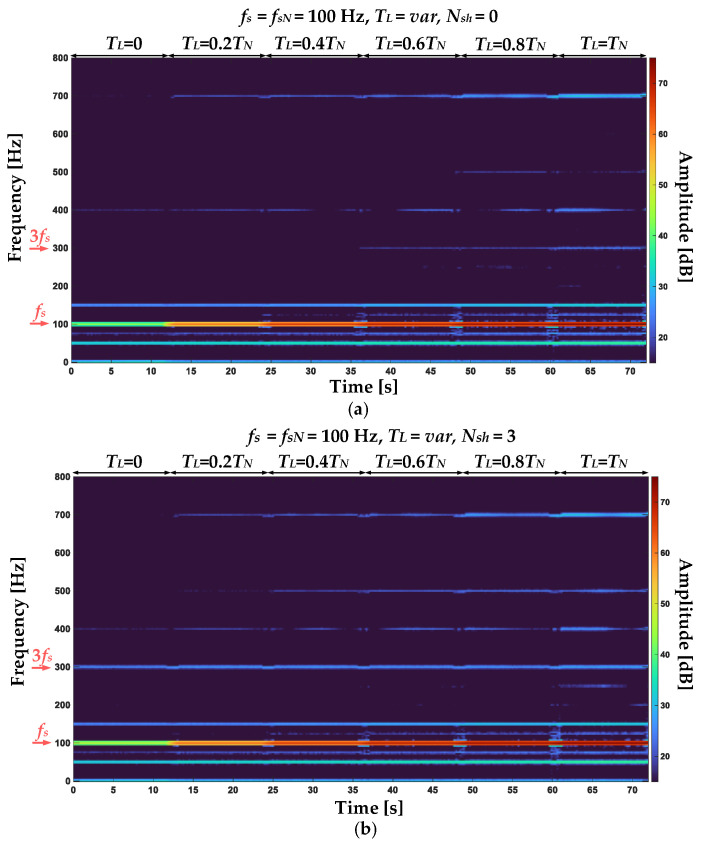
STFT spectrograms of the positive sequence stator current component for (**a**) undamaged stator winding and (**b**) 3 shorted turns in phase A (*T_L_* = var, *f_s_* = 100 Hz).

**Figure 8:**
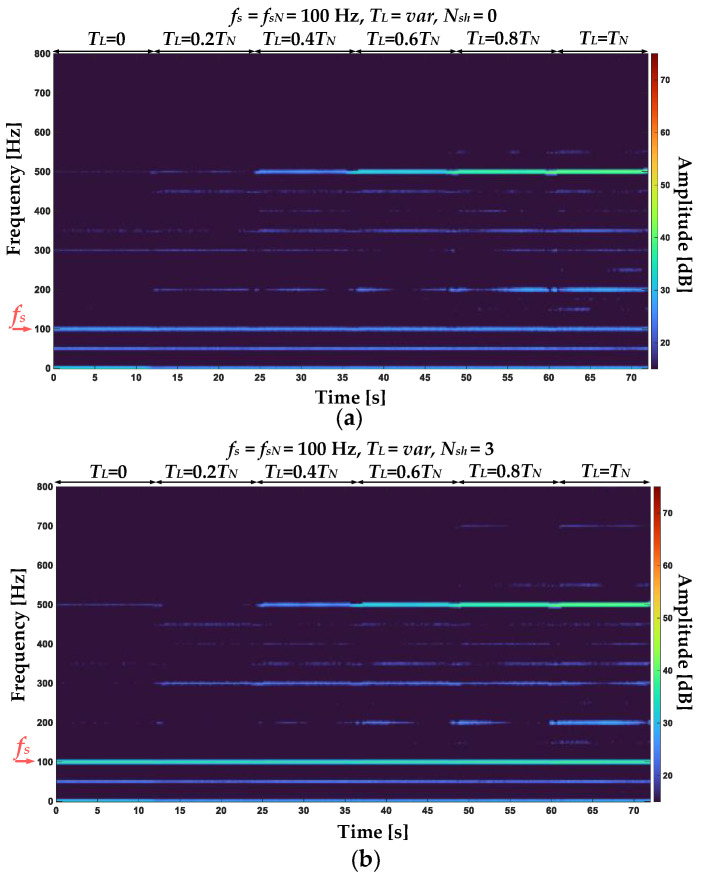
STFT spectrograms of the negative sequence stator current component for (**a**) undamaged stator winding and (**b**) 3 shorted turns in phase A (*T_L_* = var, *f_s_* = 100 Hz).

The STFT spectrograms of the positive sequence component of the stator phase currents for an undamaged motor and with ITSC of three turns in phase A of the stator winding are shown in Figure 7a and Figure 7b, respectively. The spectrograms show a significant increase in the amplitude value of the frequency component corresponding to the first harmonic (*f_s_* = 100 Hz) with the increasing load torque level. There is also a noticeable increase of the 3rd harmonic (3*f_s_* = 300 Hz) amplitude value as a result of the stator winding fault. Figure 8a,b show the STFT spectrograms of the negative sequence component of the stator phase currents for the undamaged winding and for three shorted turns in phase A, respectively. By comparing the spectrograms, a significant increase in the amplitude value of the *f_s_* frequency component can be observed as a result of a short circuit.

To emphasize the main advantage of the STFT analysis—the possibility of the harmonic tracking of the faulty components during the on-line operation of the drive system, and also to compare the sensitivity to the ITSC of the amplitudes of 3*f_s_* component in the *i_1_* spectrogram and *f_s_* component in the *i_2_* spectrogram, the experimental tests for momentary short circuits (lasting 1÷2s) and increasing *T_L_* are conducted. 

**Figure 9:**
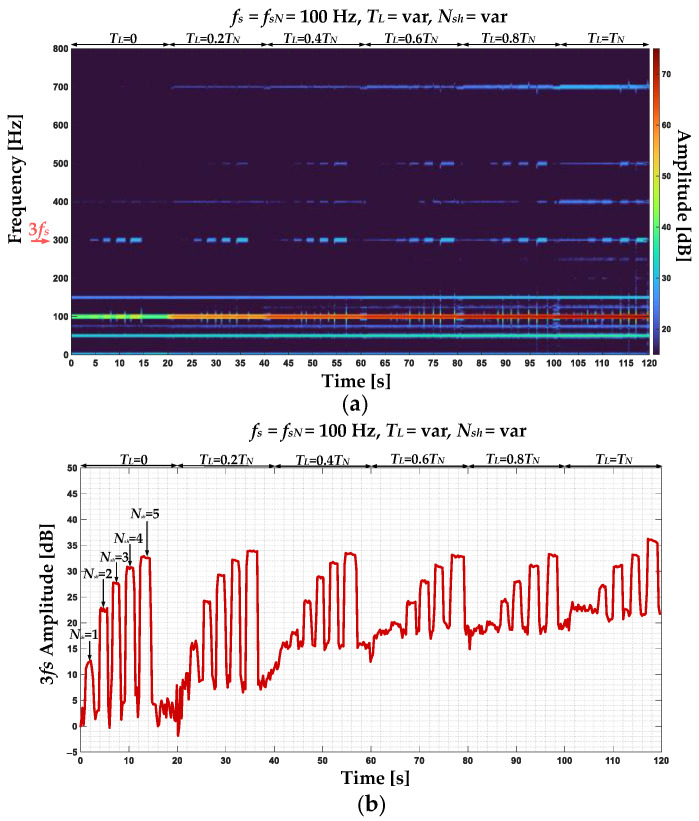
The stator phase currents positive sequence component (**a**) STFT spectrogram and (**b**) 3*f_s_* component amplitude changes during the online operation of the drive system and cyclic momentary short-circuiting of 1 to 5 turns at variable load torques.

In Figure 9, the stator phase currents positive sequence component STFT spectrogram (Figure 9a) and amplitude changes of the 3*f_s_* component during the online operation of the drive system and cyclic momentary short-circuiting of 1 to 5 turns at variable load torques (Figure 9b) are shown. In this scenario, the *T_L_* value is increased from 0 to *T_N_* with 0.2*T_N_* step and for each value, the ITSCs are performed. Based on the analysis of the results presented in this figure, it can be concluded that the value of the 3*f_s_* component amplitude increases with the increasing degree of stator winding fault (*N_sh_*). However, as the *T_L_* increases, the amplitude increase is lessened. For the rated load (*T_L_* = *T_N_*), the increase for one shorted turn is no longer visible. This is a significant limitation.

**Figure 10:**
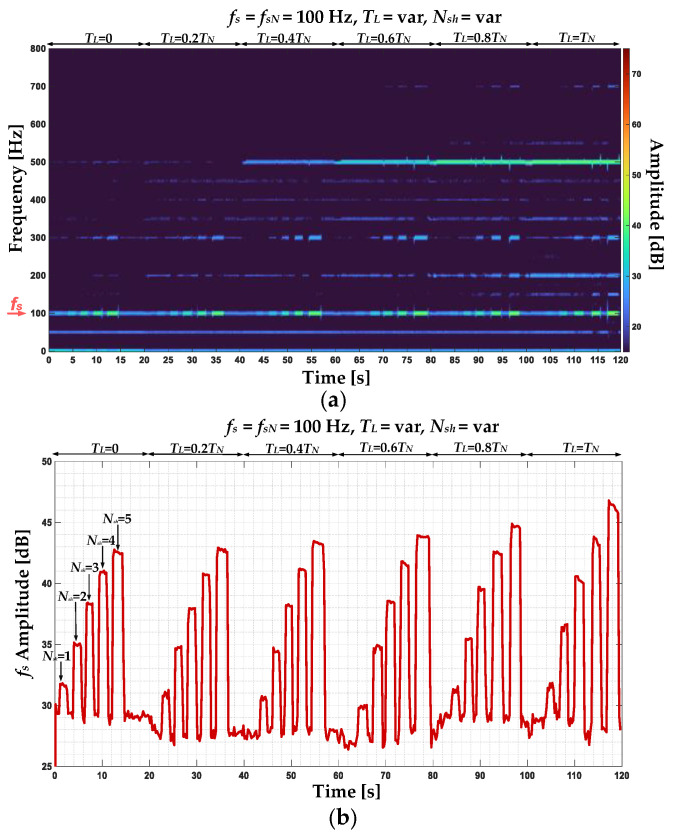
The stator phase currents negative sequence component (**a**) STFT spectrogram and (**b**) *f_s_* amplitude changes during the online operation of the drive system cyclic momentary short-circuiting of 1 to 5 turns at variable load torques.

In Figure 10, the stator phase currents negative sequence component STFT spectrogram and the *f_s_* component amplitude changes during the online operation of the drive system and the same operating conditions and stator winding states as presented for positive sequence component analysis are shown. In this case, the *f_s_* component amplitude increases as a result of the stator winding fault in the entire range of the analyzed working drive system operating conditions. The increase is visible also for the incipient stage of the fault—for one shorted turn (*N_sh_* = 1). Therefore, an increase in the amplitude of this frequency component is a good indicator of the ITSC fault.

In order to assess the exact impact of the ITSC on the amplitude of a given frequency component and the subsequent comparison of the increases between the fault indicators extracted from positive and negative stator phase current symmetrical components, the increase in the amplitude for a given *N_sh_* in relation to the value for an undamaged motor is analyzed:

(7) $$A_{DIFF}(f_{c})=A_{Damaged}(f_{c})-A_{Undamaged}(f_{c})$$

where *f_c_* is the characteristic failure frequency component and *A_Damaged_* and *A_Undamaged_* are the amplitudes of the *f_c_* component for an damaged and undamaged motor, respectively.

**Figure 11:**
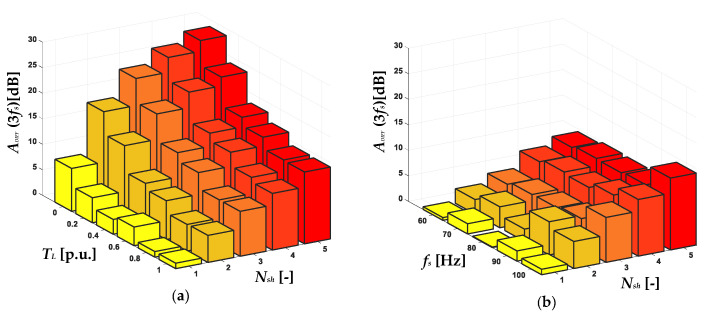
The impact of *N_sh_* in the PMSM stator winding and (**a**) *T_L_* and (**b**) *f_s_* value on the increase in amplitude of 3*f_s_* frequency component in the STFT spectrogram of the positive sequence component of the stator phase currents.

**Figure 12:**
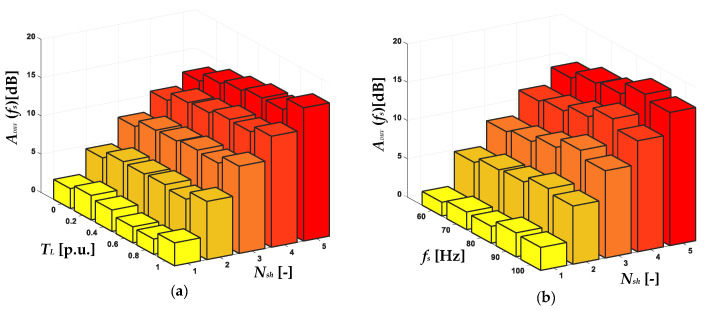
The impact of *N_sh_* in the PMSM stator winding and (**a**) *T_L_* and (**b**) *f_s_* value on the increase in amplitude of *f_s_* frequency component in the STFT spectrogram of the negative sequence component of the stator phase currents.

The influence of the stator winding fault degree (*N_sh_*) and *T_L_* on the increase of the amplitude of the 3*f_s_* frequency component in the positive sequence component spectrogram is shown in Figure 11a. The dependence on the *f_s_* value is illustrated in Figure 11b. On the basis of the presented results, it can be concluded that the increase in amplitude of 3*f_s_* caused by the stator winding fault is significant, especially in the case of the motor operating at a rotation speed close to the rated value. Nevertheless, as the value of the *f_s_* decreases, the fault sensitivity (*A_DIFF_*) is much lower. The same trend is visible for the increasing level of *T_L_*, which was also mentioned in the analysis of the results presented in Figure 9. In the case of the *f_s_* component amplitude increases in the negative sequence spectrogram (Figure 12), this limitation does not occur. The increase caused by the ITSC is visible in the entire range of the analyzed *T_L_* levels and the frequencies of the supply voltage *f_s_*, as is also the case in the early stage of damage for one shorted turn.

The results presented above and also thorough analysis of changes in harmonic amplitudes visible in the STFT spectrograms, caused by the stator winding faults, allowed for concluding that the component most sensitive to the ITSC is the *f_s_* amplitude in the spectrogram of the negative sequence stator phase currents component.

## 6. Machine Learning-Based Stator Winding Fault Classifiers

In this research, the automatization of the PMSM stator winding fault classification process based on the selected ML algorithms is proposed. The models under analysis are SVM, NB and MLP. This subsection covers the theoretical foundations and training process of these models. The possibility of improving the accuracy of these algorithms by tuning the hyperparameters is also presented.

### 6.1. Theoretical Foundations

#### 6.1.1. SVM

**Figure 13:**
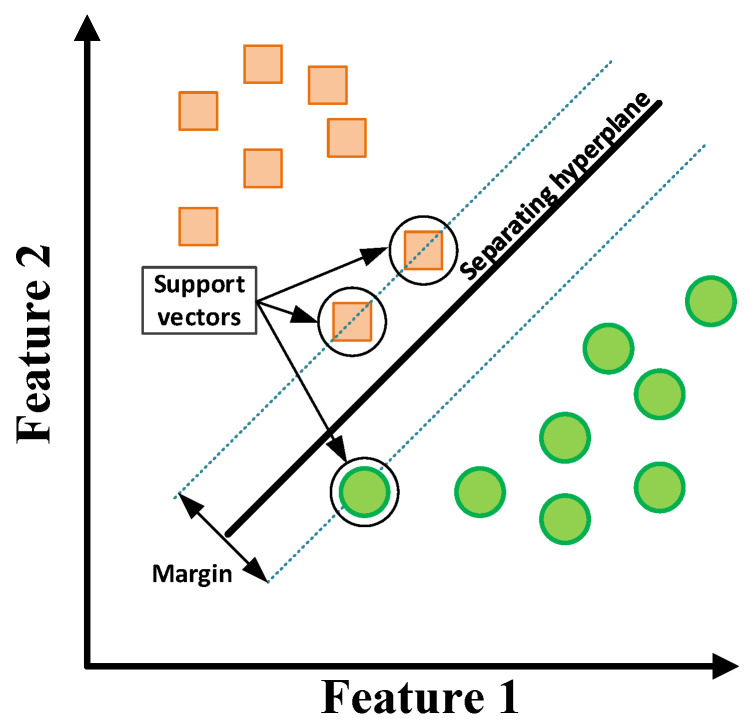
SVM classification principle.

SVM is an ML algorithm that is widely used in various classification problems. It was proposed in 1999 by Vladimir Vapnik [47]. The principle of SVM classification is to find the hyperplane that separates data points belonging to one class from points belonging to another class as much as possible in order to maximize the margin. This concept is shown in Figure 13.

Given data input **x***_i_* (*i* = 1, 2, …, *N_s_*), where *N_s_* is the number of samples, the samples are assumed to have two classes (binary classification), namely a positive class and a negative class. Each class is associated with a label: *y_i_* = 1 for the positive class and *y_i_* = −1 for the negative class. In the case of the linearly separable data, it is possible to determine the hyperplane that separates the given data [48]:

(8) $$f(x)=w^{T}x+b=\sum_{j=1}^{N_{s}}w_{j}x_{j}+b=0,$$

where **w** is the *N_s_*-dimensional normal vector of the hyperplane and *b* is a scalar.

In the linearly separable case, in order to find the optimal hyperplane with the maximum classification margin, it is necessary to solve the following optimization problem [48,49]:

(9) $$\underset{w,b}{min}\frac{||w|{|}^{2}}{2},$$

is subject to:

(10) $$y_{i}(w^{T}x_{i}+b)\geq 1,\;i=1,2,...N_{s}.$$

Taking into account the linearly inseparable data, the optimal hyperplane separating data can be obtained as a solution to the following optimization problem [47,48]:

(11) $$\underset{w,b}{min}(\frac{||w|{|}^{2}}{2}+C\sum_{i=1}^{N_{s}}\xi_{i}),$$

is subject to:

(12) $$\left\{\begin{array}{ll} y_{i}(w^{T}x_{i}+b)\geq 1-\xi_{i},\; & i=1,2,...,N_{s}\\ \xi_{i}\geq 0,\; & i=1,2,...,N_{s} \end{array}\right.$$

where *ξ_i_* is the distance between the margin and *C* is the error penalty. 

To solve this optimization problem, by introducing the Lagrange multiplier *α_i_* > 0 and subject to (12), the dual quadratic optimization problem is obtained [47,48]:

(13) $$\underset{\alpha }{max}\;L(\alpha )=\sum_{i=1}^{N_{s}}\alpha_{i}-\sum_{i,j=1}^{N_{s}}\alpha_{i}\alpha_{j}y_{i}y_{j}x_{i}x_{j},$$

is subject to:

(14) $$\alpha_{i}\geq 0,\;i=1,2,...,N_{s},\sum_{i=1}^{N_{s}}\alpha_{i}y_{i}=0.$$

To make the linear classification possible, inner multiplication in (13) is replaced by the kernel function. The kernel functions transform the training data set, so that a nonlinear decision surface can be transformed into a linear equation in a higher dimension space. Then, the decision function is defined as follows [47,48]:

(15) $$f(x)=sign(\sum_{i,j=1}^{N_{s}}\alpha_{i}y_{j}K(x_{i},x_{j})+b)$$

There are different kernel functions used in SVMs, such as linear, polynomial and Gaussian. Choosing an appropriate kernel function is very important because the kernel defines a feature space in which the training set examples will be classified [48]. In this research linear, polynomial and Gaussian kernel functions are verified. The most popular kernel functions are defined as follows:

(16) $$\;K_{Linear}(x_{1},x_{2})=x_{1}^{T}x_{2},$$

(17) $$K_{Gaussian}(x_{1},x_{2})=e^{-(\frac{||x_{1}-x_{2}|{|}^{2}}{2\sigma^{2}})},$$

(18) $$K_{Polynomial}(x_{1},x_{2})={\left(x_{1}^{T}x_{2}+1\right)}^{\gamma },$$

where *σ* is the width of the Gaussian function and *γ* is the degree of the polynomial. 

The initial version of the SVM algorithm discussed above was used to perform a binary classification—distinguishing between two classes only. In real-world problems, more than two classes can be classified, such as different types of faults in the fault diagnosis field. Therefore, multi-class classification techniques are applied [48]. They allow for categorizing the test data into multiple class labels included in the training dataset. In this research, the One-vs-One multiclassification method is used. 

**Figure 14:**
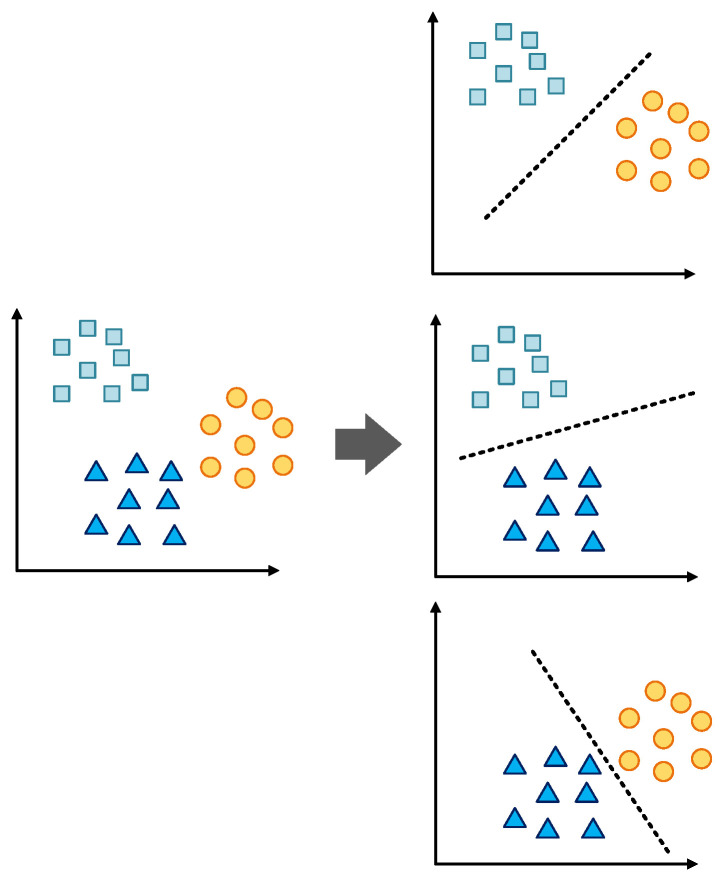
One-vs-one multiclass classification technique principle.

Let *N_C__L_* > 2 be the number of classes. The One-vs-One approach constructs *N_C__L_*(*N_C__L_*−1)/2 binary classifier models. An example of this classification for *N_C__L_* = 3 is shown in Figure 14. The multi-class classification problem is divided into three binary classification problems. The more detailed mathematical foundations of this algorithm are discussed in [50].

#### 6.1.2. NB

Due to its simplicity, efficiency and efficacy, the NB algorithm continues to be one of the top 10 algorithms in the machine learning community [50]. NB models are commonly used for classification problems. The operation principle of the NB classifier is based on Bayesian theory. It assumes that each feature of a particular class is irrelevant to other features. The NB is a probabilistic classifier. Assuming *A*_1_, *A*_2_, …, *A_m_* are *m* attributes, given a test instance *x* represented by an attribute value vector [*a*_1_; *a*_2_;…; *a_m_*], NB predicts the class label of a new instance *x* using the following equation [51]:

(19) $$c(x)=\arg\underset{c\in C}{\max}P(c)\prod_{j=1}^{m}P(a_{j}|c)$$

where *c*(*x*) is the class label of the test instance *x* predicted by NB, *a_j_* is the value of the *j*-th attribute *A_j_* and *C* is the collection of all possible class labels *c*. 

There are different types of Bayesian classifiers. They are divided according to the method of calculating the conditional probability *P*(*a_j_*|*c*). One of the most popular classifiers is the Gaussian Naïve Bayes (GNB). For GNB, the probability is calculated as follows:

(20) $$P(a_{j}|c)=\frac{1}{\sqrt{2\pi \sigma_{c}{}^{2}}}\exp-(\frac{{\left(a_{j}-\mu_{c}\right)}^{2}}{2\sigma_{c}{}^{2}})$$

The Kernel Naïve Bayes (KNB) is also widely used in the data classification field. More detailed theoretical foundations connected with the NB algorithm can be found in [50,51]. 

#### 6.1.3. MLP

MLP is one of the most popular types of neural networks that are utilized in the field of electrical motor fault diagnosis. Compared to the other structures of neural networks, MLPs are relatively easy to implement in embedded systems. MLPs are feedforward neural networks. They consist of the input layer, one or more hidden layers and the output layer. Each neuron in each layer is connected to a neuron in the next layer. 

**Figure 15:**
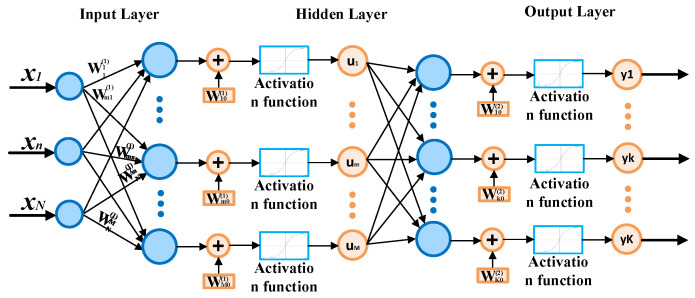
MLP exemplary structure.

The structure of the MLP network has a direct impact on the effectiveness of the model. The key parameters to be set in the process of the NN model design are the number of layers, number of neurons in each layer and type of activation function. Equation (21) describes the output of an exemplary two-layer MLP network. The exemplary structure of this type of network is presented in Figure 15 [52].

The training process of the MLP model is based on the modification of the weights to minimize the objective function [52]. The two-layer MLP output signal is described as follows [39]:

(21) $$y_{k}=f^{\left(2\right)}(\sum_{m=1}^{M}w_{km}^{\left(2\right)}f^{\left(1\right)}(\sum_{n=1}^{N}w_{mn}^{\left(1\right)}x_{n}+w_{m0}^{\left(1\right)})+w_{k0}^{\left(2\right)}),$$

where *x_n_*—*n*-th value of the input, *y_k_*—output value of the *k*-th neuron, *f*^(1)^, *f*^(2)^—activation function of the 1st and 2nd layer and *w*—weight of the neuron in the selected layer. 

### 6.2. Training Process and Offline Verification

Based on the fault indicators extracted with the use of the symmetrical components stator current STFT analysis, the input vector of the ML models consists of the amplitudes of the *f_s_* components in the positive (*Af_si1_*) and negative (*Af_si2_*) sequence components spectrograms, as well as the determined *f_s_* value: *X* = [*Af_si1_*, *Af_si2_*, *f_s_*]. The rationale for selecting these elements of the input vector is as follows: *Af_si1_* changes along with the *T_L_* changes, so it makes the models robust to the load changes, and *Af_si2_* is very sensitive to the ITSC fault and *f_s_* is easy to determine and makes the models independent of the *f_s_* value. 

The collected dataset consists of 1000 vectors, 70% of which are used in the training process, and the remaining 30% are used for offline tests. These vectors correspond to different states of the stator winding (*N_sh_* = {0; 1; 2; 3; 4; 5}) and motor operating conditions (*T_L_* = {0; 0.2*T_N_*; 0.4*T_N_*; 0.6*T_N_*; 0.8*T_N_*; *T_N_*}, *f_s_* = {80 Hz, 90 Hz, 100 Hz}). The database was collected during the experiments carried out on the described experimental setup.

In the following subsections, the training process, hyperparameters tuning and offline test results of the proposed ML-based fault classifier models are presented. The accuracy of these models is compared for different parameters and the best one for each algorithm is selected. 

The model accuracy defines how often predictions equal actual (true) labels. This metric indicates how accurate the developed model is in the tasks of the ITSC classification and is defined as follows:

(22) $$\mathrm{Accuracy}=\frac{n_{actual}}{N_{t}}\cdot 100\%$$

where *n_actual_* represents the number of input vectors that the selected ML model is able to classify correctly and *N_t_* is the total number of vectors in the training set.

#### 6.2.1. SVM

**Figure 16:**
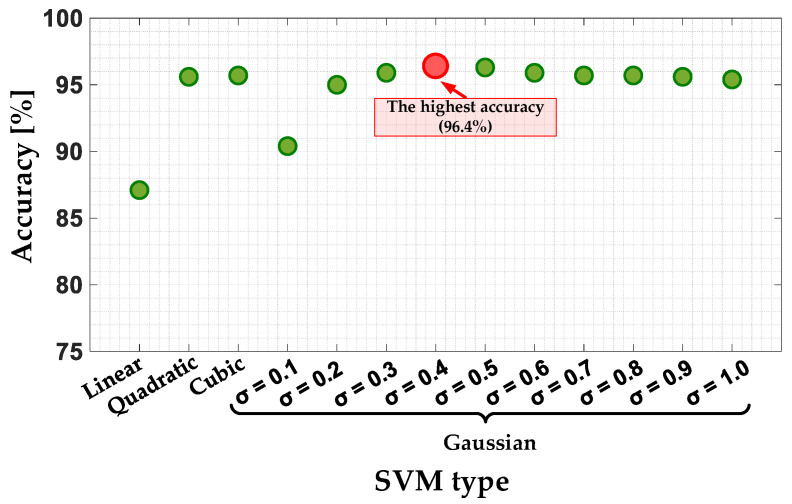
The impact of the SVM type on the SVM classifier accuracy.

In the design process of the SVM classifier model, it is necessary to select the appropriate kernel function. The kernel functions analyzed are Linear, Quadratic, Cubic and Gaussian function with different width. The accuracy of the model for selected cases analyzed is presented in Figure 16 and grouped in Table 1. Based on these results, it can be concluded that the highest model accuracy (96.4%) is achieved for the Gaussian kernel function with the width of σ = 0.4.

In order to evaluate the effectiveness of the proposed stator winding fault classifiers in offline tests, and in the next stage, in online tests, the *C_EFF_* index is introduced. It determines the ratio of the correctly classified stator winding states to the sum of the correct classifications and misclassifications. This index is defined by the following equation:

(23) $$C_{EFF}=\frac{Y_{C}}{Y_{C}+Y_{M}}\cdot 100\%,$$

where *Y_C_*—number of correct stator winding state classifications and *Y_M_*—number of stator winding state misclassifications performed by the analyzed ML models.

**Figure 17:**
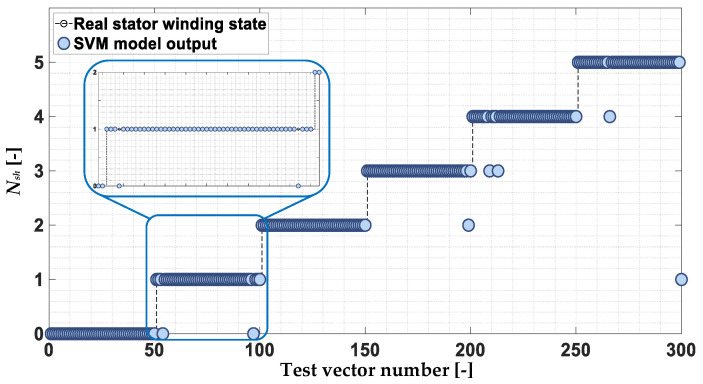
Response of the SVM stator winding fault classifier response to the test data set.

The response to the test vectors is shown in Figure 17. In this case, the effectiveness achieved by the SVM-based ITSC failure classifier is as high as 97.7%. There are only single misclassifications of the real PMSM stator winding state.

#### 6.2.2. NB

**Figure 18:**
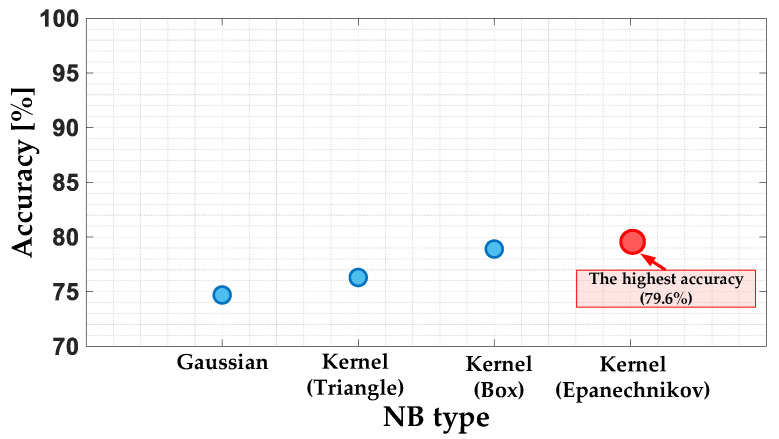
The impact of the NB type on the NB classifier accuracy.

The NB models are characterized by the function that calculates the conditional probability. The compared types of the NB classifiers are Gaussian (GNB) and Kernel Naïve Bayes (KNB) with triangle, box and Epanechnikov kernel. The accuracy of the model for each type of NB classifier is shown in Figure 18 and grouped in Table 2. Based on this, it can be concluded that the highest model accuracy (79.6%) is achieved for the KNB with the Epanechnikov kernel function. The accuracy of this model is much lower than for the model based on the SVM algorithm.

**Figure 19:**
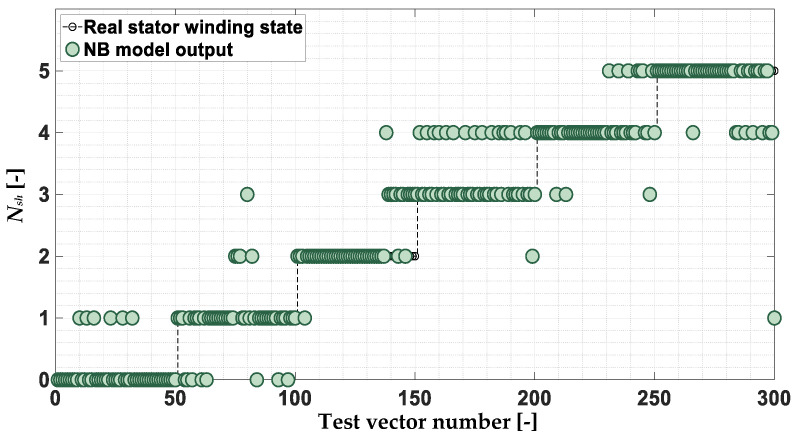
Response of the NB stator winding fault classifier to the test data set.

The response of the NB model to the test vectors is shown in Figure 19. The effectiveness of the PMSM stator winding failure classifier based on the NB model for this verification is equal to 77.7%. Misclassification is also visible when distinguishing between an undamaged motor (*N_sh_* = 0) and an early stage of damage (*N_sh_* = 1). This is a significant limitation, as the goal of the research is to detect the PMSM stator winding fault as soon as possible—at a very early stage of damage.

#### 6.2.3. MLP

In this investigation, the MLP structure is selected based on the constructivist approach, i.e., gradually adding neurons in hidden layers and verifying the model accuracy. The hyperbolic tangent sigmoid activation function is applied to the activation functions and the Levenberg–Marquardt gradient algorithm is used for training purposes.

**Figure 20:**
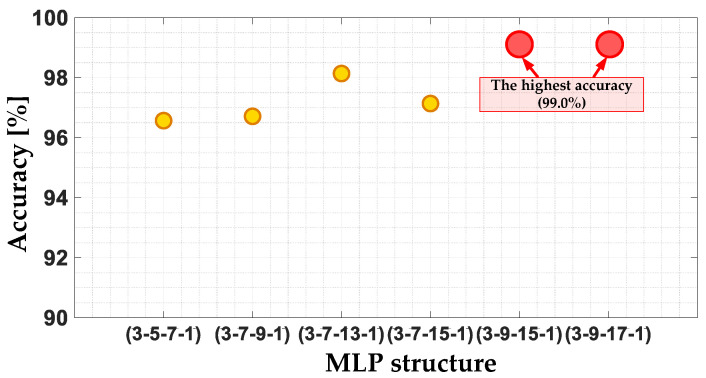
The impact of the MLP structure on the MLP classifier accuracy.

Figure 20 shows the accuracy of the MLP model for the selected network structures from those that have been tested. Based on this comparison, it can be concluded that the highest model accuracy (99.0%) is achieved for two MLP structures. The first one contains two hidden layers with 9 and 15 neurons and the second one with 9 and 17 neurons. The first of these models is used in further tests because of its simpler structure. The model accuracies for each of the tested structures are grouped in Table 3.

**Figure 21:**
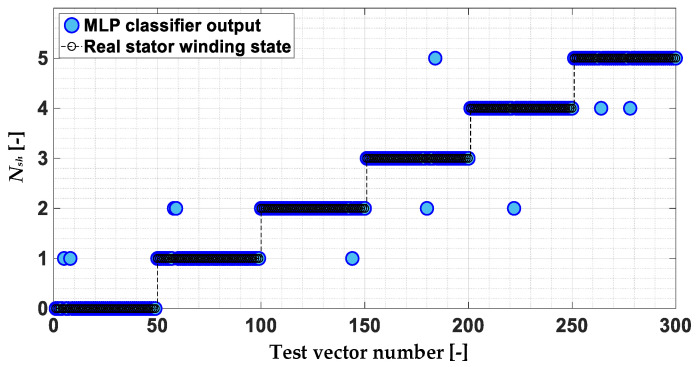
Response of the MLP stator winding fault classifier to the test data set.

The response of the MLP model to the test vectors, taking into account the rounding of the network output to the nearest integer, is shown in Figure 21. The effectiveness of the PMSM stator winding failure classifier based on the MLP model for this verification equals 97.8%.

### 6.3. Summary

**Figure 22:**
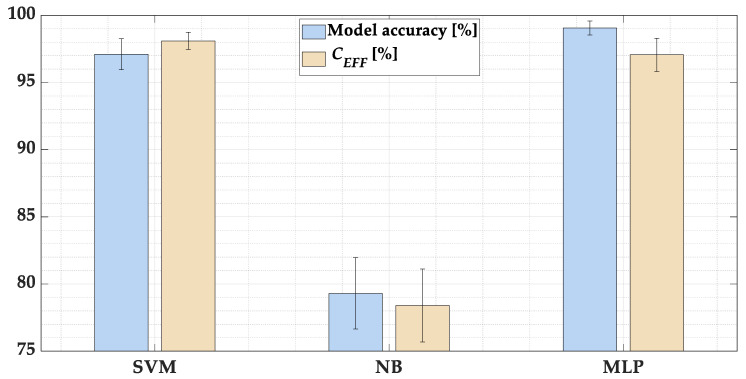
The average accuracy and *C_EFFs_* together with the standard deviations of the analyzed models.

Comparison of the effectiveness of the analyzed ML-based classifier models for various hyperparameters allowed for selecting the models with the highest effectiveness in the classification of the PMSM stator winding condition. The average accuracies and *C_EFFs_* together with the standard deviations in five trials (for different partition of training and test data) for these models are presented in Figure 22 and grouped in Table 4. Both the accuracy and the *C_EFF_* for the test vectors are significantly higher for the SVM- and MLP based stator winding fault classifiers, compared to the NB-based model. In the next step, online tests will be carried out in order to finally evaluate the classifier models.

## 7. Concept and Online Verification of the Intelligent Diagnosis System of the PMSM Stator Winding Faults

**Figure 23:**
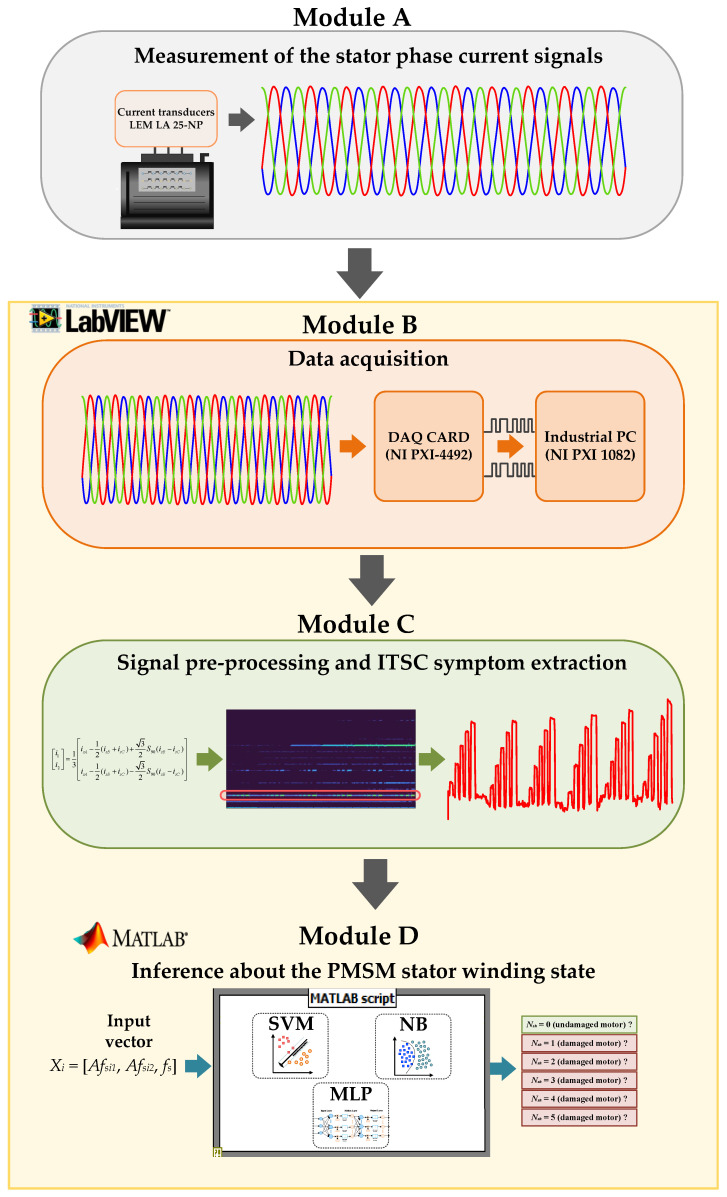
Structured flowchart of the intelligent diagnosis system of the PMSM stator winding faults concept.

In this section, the proposal and online verification of the original intelligent PMSM stator winding fault diagnosis system developed in LabVIEW and MATLAB programming environment is presented. The structured flowchart of this system is shown in Figure 23. It consists of four main modules: (1) Measurement of the stator phase current signals, (2) Data acquisition, (3) Signal pre-processing and ITSC symptom extraction and (4) Inference about the PMSM stator winding state. The functionality of each of these modules is described in Table 5.

**Figure 24:**
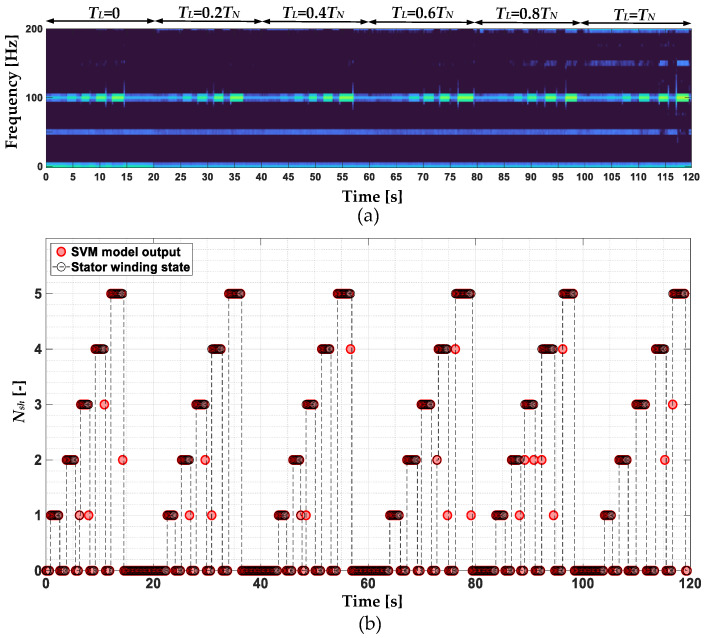
(**a**) Part of the stator phase current negative sequence component STFT spectrogram and (**b**) response of the SVM-based ITSC fault classifier.

**Figure 25:**
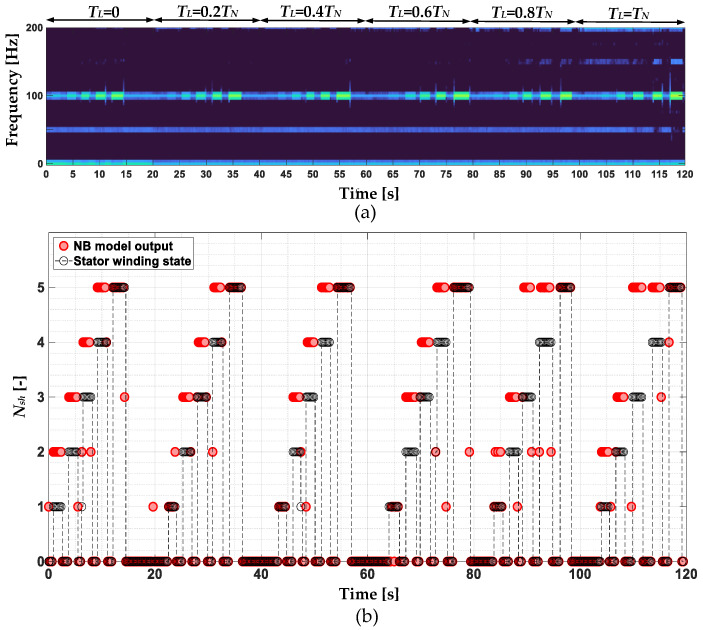
(**a**) Part of the stator phase current negative sequence component STFT spectrogram and (**b**) response of the NB-based ITSC fault classifier.

**Figure 26:**
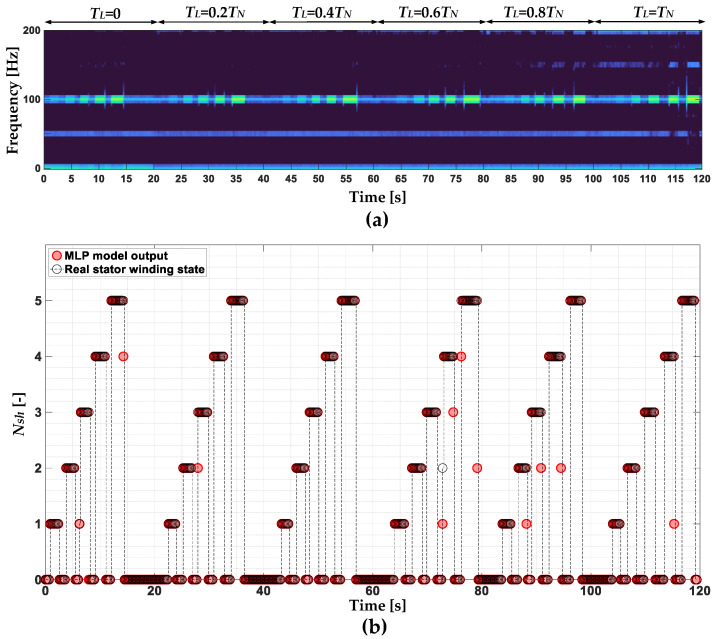
(**a**) Part of the stator phase current negative sequence component STFT spectrogram and (**b**) response of the MLP-based ITSC fault classifier.

In order to finally assess the effectiveness of the stator winding fault classifiers, their effectiveness is verified during online operation of the drive system. The online operation of the classifiers is verified in the test during which one to five turns in phase A are shorted for about 1–2 s, successively increasing the load torque with a step of 0.2*T_L_*, up to the rated value. Parts of the negative-sequence STFT spectrogram showing the changes of the *f_s_* frequency component, as well as the responses of the SVM, NB and MLP classifiers, are shown in Figure 24, Figure 25 and Figure 26, respectively. 

The highest *C_EFF_* index of 96.1% is achieved by the SVM-based PMSM stator winding fault classifier. The NB model achieved a significantly lower effectiveness, amounting to 67.4%. The MLP model achieved a slightly lower effectiveness, amounting to 97.8%. Therefore, it can be stated that the SVM- and MLP- based models can be successfully applied in the last module (D) of the developed stator current data-driven intelligent stator winding fault diagnosis system. The effectiveness achieved by the fault classifier based on the NB algorithm is insufficient for the implementation of effective diagnostics. The relatively low effectiveness of the NB-based classifier may be caused by the fact that the operation principle of this algorithm assumes that each feature of a particular class is irrelevant to other features. In the case of fault diagnostics and the proposed input vector, its elements are interrelated.

## 8. Conclusions

This paper proposes an original intelligent PMSM stator winding fault diagnosis system based on the hybrid method that combines the STFT analysis of the stator phase current symmetrical components and selected ML algorithms. On the basis of the conducted experimental tests, it is proven that the amplitude value of fundamental frequency component (*f_s_*) in the STFT spectrogram of the negative sequence component of the stator phase current is a very good indicator of PMSM stator winding fault. This indicator is susceptible to ITSC in a wide range of tested motor operating conditions and for the incipient stage of the fault.

The effectiveness of the automatic inference about the state of the PMSM stator winding has been verified for three ML-based models: SVM, NB and MLP. The input vector of these models was built based on an in-depth analysis of the STFT results. Experimental studies have shown that the SVM- and MLP-based PMSM stator winding faults classifier are characterized by much higher model accuracy and classification effectiveness compared to the NB. A significant influence of the model key parameters on their accuracy has also been proven. It is shown that the model design process should be carried out carefully.

The proposed stator phase current data-driven intelligent stator winding fault diagnosis system has potential for real deployment in the industry. This system has a modular structure, which allows it to be scalable and easy to modify. The proposed methodology can be improved to detect other motor faults by the adaptation of the ML for more advanced multi-fault-classification task.

Further research will focus on the development of a low-cost diagnostic system based on the proposed concept, but with the use of less expensive components. An attempt will be made to implement the described software in one of the low-budget microcontrollers. In addition, to evaluate the possibility of using information from several, different ML-based models in parallel in order to improve the effectiveness of the entire system through the use of ensemble learning technique is being planned.

## Figures and Tables

**Table 1:** The SVM classifier accuracy for different kernel function.

Kernel Function	Accuracy	σ [-]
Linear	87.1%	-
Quadratic	95.6%	
Cubic	95.7%	
Gaussian	90.4%	0.1
	95%	0.2
	95.9%	0.3
	**96.4%**	**0.4**
	96.3%	0.5
	95.9%	0.6
	95.7%	0.7
	95.7%	0.8
	95.6%	0.9

**Table 2:** The NB classifier accuracy for different conditional probability.

NB Classifier Type	Accuracy
GNB	74.7%
KNB (Triangle)	76.3%
KNB (Box)	78.9%
**KNB (Epanechnikov)**	**79.6%**

**Table 3:** The MLP classifier accuracy for different structures of the network.

MLP Structure	Accuracy
3-5-7-1	96.6%
3-7-9-1	96.7%
3-7-13-1	98.1%
3-7-15-1	97.1%
**3-9-15-1**	**99.0%**
3-9-17-1	99.0%

**Table 4:** The comparison of the average accuracy and *C_EFF_* with standard deviation of the analyzed ML based classifiers.

Average	ML-Based Classifier		
	SVM	NB	MLP
Accuracy [%]	97.1 (±1.16)	79.3 (±2.65)	**99.0 (±0.52)**
*C_EFF_* [%]	**98.1 (±0.65)**	78.4 (±2.72)	97.0 (±1.23)

**Table 5:** The details of the intelligent diagnosis system modules.

Module	Details
A	The first module (A) is responsible for the measurement of the diagnostic signal. In this research, the stator phase current signals are used. These signals are measured with the use of the multirange current transducers (LEM LA 25-NP).
B	The main task of the second module (B) is the stator phase current signal acquisition. It is realized by the DAQ PXI-4492 measurement card by NI, placed in the industrial PC (NI PXI 1082). From the software point of view, the link between the DAQ card and the diagnosis system is realized with the use of the DAQ assistant block available in the LabVIEW environment.
C	The third module (C) plays a key role in the structure of the developed PMSM stator winding fault diagnosis system. It is responsible for the symmetrical current components calculation, their STFT analysis and also for the symptoms (amplitudes of the characteristic frequency components) extraction. In addition, the online tracking of the selected amplitudes *Af_s1_*, *Af_s2_* and the determination of power supply frequency *f_s_* is realized here.
D	The last module (D) performs the function of automatic inference about the PMSM stator winding state. The input vector elements from module C are transferred to the ML-based pre-trained models (SVM, NB and MLP). These models are developed with the use of the *Statistics and Machine Learning Toolbox* that is available in the MATLAB environment. The combination of the MATLAB models with the virtual diagnostic system developed in LabVIEW is realized using the MATLAB script structure available in LabVIEW. The classified state of the stator winding is generated at the model output.

## Data Availability

Not applicable.

## Appendix A

**Table A1:** Rated parameters of the tested PMSM.

Name of the Parameter	Symbol	Units	
Power	*P_N_*	2500	[W]
Torque	*T_N_*	16	[Nm]
Speed	*n_N_*	1500	[r/min]
Stator phase voltage	*U_sN_*	325	V
Stator current	*I_sN_*	6.6	[A]
Frequency	*f_sN_*	100	[Hz]
Pole pairs number	*p_p_*	4	[-]
Number of stator turns	*N_st_*	2 × 125	[-]
